# Efficacy of deep transcranial magnetic stimulation with H7 coil in gambling disorder: COMET-7 study protocol

**DOI:** 10.1192/j.eurpsy.2026.10899

**Published:** 2026-07-31

**Authors:** D. Bodor, K. Matić, S. Vuk Pisk, K. Bosak, V. Grošić, E. Ivezić, M. Grah, K. Laškarin, T. Gajšak, Ž. Milovac, Ž. Bajić, I. Simunovic Filipcic, I. Filipčić

**Affiliations:** 1 University Psychiatric Clinic Sveti Ivan, Zagreb; 2 Faculty of Dental Medicine and Health, Josip Juraj Strossmayer University of Osijek, Osijek; 3 University of Applied Health Science; 4 Department of psychiatry and psychological medicine, University Hospital Centre Zagreb, Zagreb, Croatia

## Abstract

**Introduction:**

Gambling disorder(GD) lacks approved pharmacotherapies and current psychological treatments face adherence and effectiveness limitations. Deep repetitive transcranial magnetic stimulation (dTMS) with the H7 coil targets medial prefrontal and anterior cingulate circuits implicated in impulsivity and compulsivity yet has not been tested for GD in a rigorous sham-controlled trial. We describe the COMET-7 protocol.

**Objectives:**

To evaluate the efficacy, safety and tolerability of high-frequency H7-coil dTMS for GD versus sham. Secondary objectives: to identify clinical/psychological predictors and heart-rate variability (HRV) correlates of response, and to estimate economic impact of this treatment.

**Methods:**

Single-centre, prospective, double-blind, parallel-group, randomised, sham-controlled trial with concealed allocation and blinded outcome assessment at the University Psychiatric Clinic Sveti Ivan. Men aged 18–64 years with ICD-10 F63.0 GD, right-handed, entering treatment are eligible. Key exclusions include psychosis, recent substance use disorders (except nicotine) and major neurological disease. Participants will be randomised 1:1 by minimisation (Pocock–Simon) on prespecified factors. The active arm receives FDA-pattern H7-coil dTMS: 20 Hz, 2-s trains, 20-s inter-train, 2,000 pulses/session at 100 % motor threshold, five sessions/week for five weeks plus four sessions in week six (total 58,000 pulses); the sham arm receives device-matched sham. All patients continue standard multidisciplinary care, held constant across arms. Outcomes are assessed at baseline and six weeks; selected measures are monitored weekly. The primary endpoint is change in Gambling Symptom Assessment Scale (G-SAS). Secondary endpoints include craving (GUS, VAS), PG-YBOCS, PGSI, SOGS, abstinence, depressive and anxiety symptoms (BDI-II, BAI), quality of life (QOLI), HRV parameters, and adverse events. The intention-to-treat analysis tests the group×time interaction using mixed-model ANCOVA with prespecified covariates. Sensitivity analyses include per-protocol and robust models. Missing outcomes in ITT are handled under a conservative no-effect assumption; covariate missingness is multiply imputed. Target sample size is 60 (30 per arm) to detect a moderate effect with 80 % power, allowing 15 % attrition. The trial will be registered on ISRCTN; ethics approval will be obtained before enrolment

**Results:**

This abstract reports the registered design, eligibility, intervention, endpoints, analysis plan and sample size to enable appraisal and replication.

**Conclusions:**

COMET-7 will provide the first double-blind, sham-controlled evidence on H7-coil dTMS for GD. By integrating clinical measures with HRV and economic evaluation, the study is designed to inform mechanistic understanding, patient selection and health-policy decisions regarding neuromodulation for behavioural addictions.

**Disclosure of Interest:**

None Declared

